# Integrated Evaluation of Age-Related Changes in Structural and Functional Vascular Parameters Used to Assess Arterial Aging, Subclinical Atherosclerosis, and Cardiovascular Risk in Uruguayan Adults: CUiiDARTE Project

**DOI:** 10.4061/2011/587303

**Published:** 2011-12-10

**Authors:** Daniel Bia, Yanina Zócalo, Ignacio Farro, Juan Torrado, Federico Farro, Lucía Florio, Alicia Olascoaga, Javier Brum, Walter Alallón, Carlos Negreira, Ricardo Lluberas, Ricardo L. Armentano

**Affiliations:** ^1^Physiology Department, School of Medicine and School of Science, CUiiDARTE, Republic University, General Flores 2125, 11800 Montevideo, Uruguay; ^2^Cardiology Department, Cardiovascular Centre, School of Medicine, CUiiDARTE, Clinical Hospital, Republic University, Avenida Italia s/n, 11600 Montevideo, Uruguay; ^3^Clinical Laboratory Department, School of Medicine, Clinical Hospital, Republic University, Avenida Italia s/n, 11600 Montevideo, Uruguay; ^4^Physics Institute, School of Sciences, CUiiDARTE, Republic University, Iguá 4225, 11400 Montevideo, Uruguay

## Abstract

This work was carried out in a Uruguayan (South American) population to characterize aging-associated physiological arterial changes. Parameters markers of subclinical atherosclerosis and that associate age-related changes were evaluated in healthy people. A conservative approach was used and people with nonphysiological and pathological conditions were excluded. Then, we excluded subjects with (a) cardiovascular (CV) symptoms, (b) CV disease, (c) diabetes mellitus or renal failure, and (d) traditional CV risk factors (other than age and gender). Subjects (*n* = 388) were submitted to non-invasive vascular studies (gold-standard techniques), to evaluate (1) common (CCA), internal, and external carotid plaque prevalence, (2) CCA intima-media thickness and diameter, (3) CCA stiffness (percentual pulsatility, compliance, distensibility, and stiffness index), (4) aortic stiffness (carotid-femoral pulse wave velocity), and (5) peripheral and central pressure wave-derived parameters. Age groups: ≤20, 21–30, 31–40, 41–50, 51–60, 61–70, and 71–80 years old. Age-related structural and functional vascular parameters profiles were obtained and analyzed considering data from other populations. The work has the strength of being the first, in Latin America, that uses an integrative approach to characterize vascular aging-related changes. Data could be used to define vascular aging and abnormal or disease-related changes.

## 1. Introduction

Atherosclerotic cardiovascular disease is a leading cause of morbidity and mortality allover the world. In general terms, atherosclerosis has a long asymptomatic latent period, which would provide an opportunity for the development of effective strategies of treatment and/or prevention. Even more, an early identification of subjects at increased risk of developing atherosclerosis would be important since prevention strategies that could have the highest impact in cardiovascular outcomes at the population level would be those instituted early. Furthermore, prevention would be more effective if tailored to individual risk.

Several methods have been proposed to stratify the cardiovascular risk, taking into account data mainly derived from European and/or North American populations. Those methods are mostly based on the identification of factors associated with the etiology and development of atherosclerosis (traditional cardiovascular risk factors). However, it is noteworthy that given the ethnic diversity in the atherosclerosis profile, the dissimilar risk associations and the different levels of genetic-environmental interactions in different populations, studies performed in a given population could not be directly applied to another (i.e., data from European people could not be extrapolated to Latin or South American). Additionally, it is noteworthy that cardiovascular risk approaches based on the assessment of conventional risk factors could not be enough to determine the cardiovascular risk of an individual [1, 2]. In this context, it has been stated that the early detection of atherosclerosis and the individual risk stratification by means of the evaluation of vascular parameters could provide a major opportunity to prevent cardiovascular events [1, 2].

Among others, central aortic pressure, pulse wave velocity, and the carotid artery arterial wall thickness and stiffness have been proposed as valuable parameters for the assessment of the individual cardiovascular risk and for the early detection of vascular damage related with the atherosclerotic disease [1–3]. Related with this, with rising frequency, the evaluation of arterial parameters (i.e., stiffness) is considered in clinical practice and in clinical studies. This is exemplified by the inclusion of the vascular evaluation in recent guidelines related with hypertension management and with cardiovascular risk assessment in asymptomatic adults [1, 2]. However, in spite of its emergence as a useful tool for risk stratification and atherosclerosis assessment, a widespread implementation of vascular evaluation has been hampered, among other factors, due to the absence of a standardized methodology of study, and due to the lack of established normal and reference values for different populations. Related with this, taking into account the complexity of age-vascular disease interaction, an important issue would be the characterization of the structural and functional vascular changes associated with aging. Such information would contribute to differentiate abnormal or disease-related vascular changes and those expected due to aging.

Latin America encompasses a wide variety of geographic, ethnic, and socioeconomic differences. Such diversity could be reflected in the prevalence/profile of the cardiovascular risk factors, atherosclerotic vascular changes and/or in normal/reference values of vascular parameters [4–7]. Considering the described value of evaluating the arterial function and structure for cardiovascular risk stratification and diagnosis, and the lack of normal and reference levels of vascular parameters defined taking into account the differences among populations, their determination is an urgent necessity, even more when heterogeneous Latin-American populations are considered.

The present study was carried out in a Uruguayan asymptomatic and healthy population to quantify several complementary vascular parameters considered markers of arterial aging and subclinical atherosclerosis and to analyze the age-associated changes in those markers, comparing the obtained data with those from other populations. To fulfill the described aims, we applied an integrative approach and we measured several complementary structural and functional vascular parameters, using noninvasive gold standard methods.

## 2. Materials and Methods

### 2.1. Study Population and Subjects Groups

CUiiDARTE Project is a population-based national study developed in Uruguay. Uruguay, with an area of approximately 176,000 square kilometers (the second-smallest nation of South America), has a population of approximately 3.5 million, of which 1.8 million live in Montevideo (Capital) and its metropolitan area. Most Uruguayans (88%) are Caucasian of European origin (mainly Spanish).

#### 2.1.1. Subject's Selection and Groups

Our study was designed to characterize, in healthy people from Uruguay, the physiological age-associated changes in arterial parameters, markers of disease. Only by knowing the physiological changes associated with age it is possible to know whether a particular value of a parameter considered marker of vascular disease is the result of normal aging or reflects a diseased arterial system. To analyze the age-related physiological changes in several parameters, a conservative approach was used and people with nonphysiological or pathological conditions were excluded. Then, we excluded (a) symptomatic subjects with or without known cardiovascular disease, (b) subjects with personal history of cardiovascular disease, (c) subjects with diabetes mellitus or renal failure, and (d) subjects with cardiovascular risk factors other than age and gender. To ensure an adequate application of the exclusion factors, we did a medical interview in which personal and family history and lifestyle habits were assessed by means of a standardized questionnaire. In addition, anthropometric and laboratory measurements (see below) were done. Subjects referred for cardiovascular evaluation in the CUiiDARTE Project were considered, employing a probability sampling strategy (cluster sampling); 388 subjects were enrolled in this study.

The subjects age range was selected in agreement with recent international consensus that recommend both, starting noninvasive arterial evaluation at ~20 years old and the development of programs for atherosclerosis screening in subjects between 45–75 years old (55–75 in women) [1, 2]. The study was approved by the Institutional Ethic Committee (Republic University) and it was conducted according to the Declaration of Helsinki and the Good Clinical Practice Guidelines. CUiiDARTE Project details can be found in: http://www.cuiidarte.fmed.edu.uy/.

Subjects were studied in a single visit. Evaluation started after 9–12 hours overnight fast. Exercise, caffeine, alcohol, and vitamin C were avoided prior (at least six hours) to the cardiovascular examination. Subjects' height and weight were measured, and the body mass index (BMI, weight to height squared ratio) calculated. Subjects with a BMI higher than 30 Kg/m^2^ were excluded. Family history of premature cardiovascular disease was defined as a first degree relative with history of cardiovascular disease at ages younger than 55 years in men and 65 years in women.

### 2.2. Laboratory Measurements

Venous blood samples were drawn and processed immediately using commercially available kits and/or laboratory methods. Procedures were standardized before the study initiation, and during the study's development they were controlled for quality by a central reference laboratory (Clinical Laboratory Department, Clinical Hospital, School of Medicine). Total cholesterol (TC) was determined by the spectrophotometry cholesterol oxidase/peroxidase enzymatic method; serum triglycerides (TG) and high density lipoprotein cholesterol (HDL-C) were determined by glycerol enzymatic method, and the precipitating reactive method, respectively. Low density lipoprotein cholesterol (LDL-C) was calculated by the Friedewald formula (LDL-C = [TC − HDL-C] − [TG/5], valid if TG <400 mg/dL) [8]. Non-HDL-C (TC − HDL-C) and TC/HDL-C were calculated.

Lipid values were classified according to NCEP-ATP III criteria [9]. Patients with a lipid profile with one or more of the following conditions: TG ≥200 mg/dL, TC ≥240 mg/dL, HDL-C <40 mg/dL, LDL-C≥ 160 mg/dL, and/or currently taking antilipemic agents were excluded at the time of the data analysis.

Subjects' anthropometric characteristics and laboratory data are shown in Table 1.

### 2.3. Noninvasive Measurements of Atherosclerotic Markers

After blood collection, subjects were taken to the laboratory for noninvasive vascular assessment. Vascular evaluation consisted in measuring, using gold-standard methodologies, complementary structural and functional vascular parameters. Ultrasonography (B-mode and Doppler), sphygmomanometry, mechanography, and applanation tonometry techniques were employed to evaluate/measure

- common (CCA), internal (ICA), and external (ECA) carotid arteries plaque presence;
- CCA intima-media thickness (CIMT) and instantaneous diameter waveforms;
- CCA stiffness (percentual pulsatility, compliance, distensibility, and stiffness index);
- aortic stiffness (carotid-femoral pulse wave velocity);
- peripheral and central (aortic) pressure levels and pulse wave analysis.

#### 2.3.1. Common (CCA), Internal (ICA), and External (ECA) Carotid Arteries Plaque Presence: CCA Intima-Media Thickness (CIMT) and Instantaneous Diameter Waveforms

Ultrasound evaluations in the CUiiDARTE Study are based on the techniques and recommendations described in international consensus [10, 11]. High-resolution B-mode carotid ultrasonography was done with a linear-array, 10 MHz transducer connected to a portable ultrasound system (MicroMaxx, Sonosite; Bothell, Wash, USA). Measurements (still images and video clips/cine loops) were digitally stored for off-line analysis. Studies were done after 10–15 minutes of recumbent rest. Before and during ultrasound examination (at 3-minutes intervals), brachial blood pressure measurements were obtained using an oscillometric device (Omron HEM-433INT Oscillometric System; Omron Healthcare Inc., Ill, USA). The average was considered as blood pressure level.

Transverse and longitudinal views from the proximal CCA to the peripheral segments of the ICA and ECA were obtained, so as to assess the presence of carotid plaques in the CCA, bulb, ICA, and ECA. Near and far walls were analyzed and images were obtained from anterior, lateral and posterior angles. A carotid plaque was defined as focal wall thickening at least 50% greater than that of the surrounding vessel, a thickening that protrudes into the lumen 0.5 mm or as a region with CIMT greater than 1.5 mm [10]. Plaque thickness was quantified at the site of maximal encroachment perpendicular to the vessel wall, and obtained by measuring (using digital calipers and automated procedures) the distance between the media-adventitia interface and the lesion surface facing the lumen.

**Figure 1:**
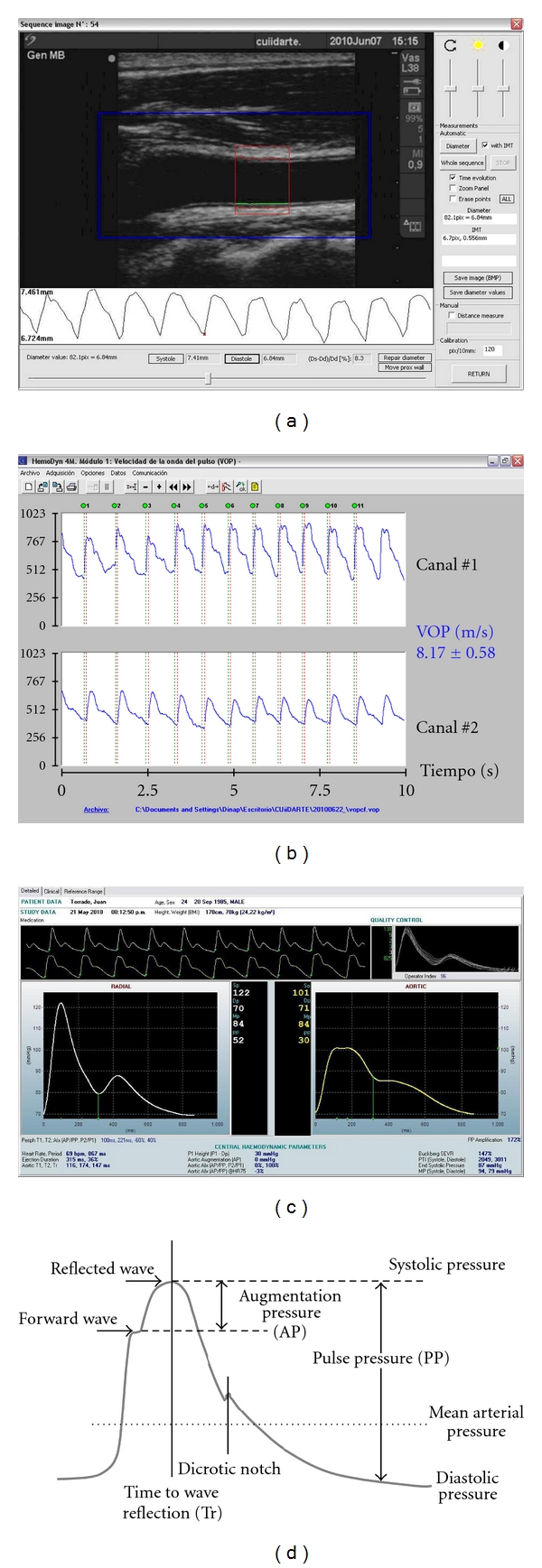
(a) Software employed to the CIMT and instantaneous diameter waveform measurement from B-Mode echographic videos. (b) Software employed to the PWV measurement from the carotid (Canal 1) and femoral (Canal 1) instantaneous waveforms. (c) Software employed to obtain the central pressure waveform (from the radial waveform recording) and to perform the peripheral and central pressure waveform analysis. (d) Schema of the pulse wave analysis (see text).

After plaque screening, longitudinal views of the CCA (obtained at the described angles), were acquired so as to measure the CIMT and to obtain the diameter waveforms. A video (cine-loop) of at least 10 seconds was recorded from each angle of interrogation. The CIMT and beat-to-beat diameter waveforms were obtained and analyzed off-line using a step-by-step border detection algorithm applied to each digitized image [12] (Figure 1(a)). A region 1.0 cm proximal to the carotid bulb was identified, and the far wall CIMT determined as the distance between the lumen-intima and the media-adventitia interfaces. If there were plaques in the area of CIMT measurement, they were included in the measurement. The used software performs multiple automated or semiautomated measurements along 1 cm and averages them, therefore increasing the accuracy of the measures. The instantaneous internal diameter (from the leading edge of the near wall intima-lumen interface to the intima-lumen interface of the far wall) waveform was obtained (Figure 1(a)).

From the vascular echographic evaluation mechanical parameters could be calculated (i.e., percentual distensibility) (Figure 1(a)). CCA wall-to-lumen ratio was quantified as (CIMT/internal diastolic diameter) ∗ 100.

#### 2.3.2. CCA Stiffness (Percentual Pulsatility, Compliance, Distensibility, and Stiffness Index)

To evaluate the CCA stiffness we calculated parameters that give complementary information about the vascular behavior. Such parameters allowed comparing our data with reported results [11]. In addition, it is noteworthy that the stiffness indexes considered are commonly used in the clinical practice, since they can be obtained just using systolic (maximal) and diastolic (minimal) diameter and/or pressure values. Percentual or fractional pulsatility (FP%) coefficient was calculated as FP% = ((SD − DD)/DD) ∗ 100, where SD and DD are systolic and diastolic internal diameters, respectively; CCA compliance (CCA_C) and distensibility (CCA_D) were quantified as CCA_C = (DS – DD)/(cSBP−cDBP) and CCA_D = ((DS − DD)/DD)/(cSBP−cDBP), where cSBP and cDBP are central systolic blood pressure and diastolic blood pressure, respectively, obtained using Applanation Tonometry (PWA, SphygmoCor, AtCor Medical Pty Ltd., Sydney, Australia). Finally, the stiffness or Beta index (*β*) was calculated as: *β* = Ln(cSBP/cDBP)/(SD − DD).

#### 2.3.3. Aortic Stiffness (Carotid-Femoral Pulse Wave Velocity)

The carotid-femoral pulse wave velocity (PWVcf) was measured to analyze aortic regional stiffness. To this end, using mechanotransducers placed simultaneously on the skin over the carotid and femoral arteries (subjects in supine position), the carotid and femoral pulse waves were recorded [11, 12] (Figure 1(b)). Straight distance between the recording sites (car-fem distance) was measured (using a tape) on the body surface. The PWVcf was automatically calculated as the quotient between the “car-fem” distance and the carotid-femoral pulse transit time difference. In agreement with international recommendations, the *real PWVcf* was calculated [3]. Real PWV is a standardized PWV, obtained if the direct “car-fem” distance and the intersecting tangent algorithm (used to detect the carotid-femoral pulse transient time) are used, and the value is multiplied by 0.8 (to reach more realistic PWV values) [3]. The reported value of PWVcf for a subject was always the average of at least 8 consecutive beats. During PWV recording brachial pressure and heart rate were quantified (Omron HEM-433INT Oscillometric System; Omron Healthcare Inc., Ill, USA).

#### 2.3.4. Peripheral and Central (Aortic) Pressure Levels and Pulse Wave Analysis

Pulse wave analysis (PWA) was used to obtain the ascending aortic pressure waveform from the radial pulse (obtained by Applanation Tonometry) using customized software (SphygmoCor 7.01, AtCor Medical, Sydney, Australia) with a previously validated generalized transfer function [13] (Figure 1(d)). The radial pulse wave was obtained with the subject seated with the arm resting on a table. The radial pulse waveform was calibrated using the diastolic and mean pressure obtained at the brachial artery (Omron HEM-433INT Oscillometric System; Omron Healthcare Inc., Ill, USA) [11].

From the aortic pulse wave, central arterial pressure was estimated, and several indexes of wave reflections were quantified from the central and peripheral waveforms: central augmentation or augmented pressure (AP), heart rate corrected (HR75) central augmentation pressure, central augmentation index (C_AGPH = (AP/PP) ∗ 100), heart rate corrected central augmentation index (C_AGPH_HR75), peripheral augmentation index (P_AIx), and the central-peripheral pulse pressure amplification ratio ((peripheral pulse pressure/central pulse pressure) ∗ 100) [14, 15] (Figure 1(c)).

### 2.4. Statistical Analysis

Based on age, the 388 subjects included (range: 18–79 years old), were divided into the following age groups: ≤20 (*n* = 74), 21–30 (*n* = 79), 31–40 (*n* = 43), 41–50 (*n* = 50), 51–60 (*n* = 59), 61–70 (*n* = 50), and 71–80 (*n* = 33) years old. Considering our aim, the Uruguayan population, the prevalence of cardiovascular disease and risk factors in the Uruguayan population, and considering an *α* = 0.05 (C.I. = 95%), the number of subjects included was enough to perform statistical inference about age-related changes in arterial parameters. We used a probability sample strategy (cluster sampling). Statistical analyses were done using Statistical Package for the Social Sciences 17.0 for Windows software. The data are expressed as the mean ± standard deviation. Proportions are presented as percentages. The correlations between the age and other parameters were determined by a linear regression analysis.

## 3. Results

### 3.1. Peripheral and Central (Aortic) Pressure Pulse Wave Analysis

**Figure 2:**
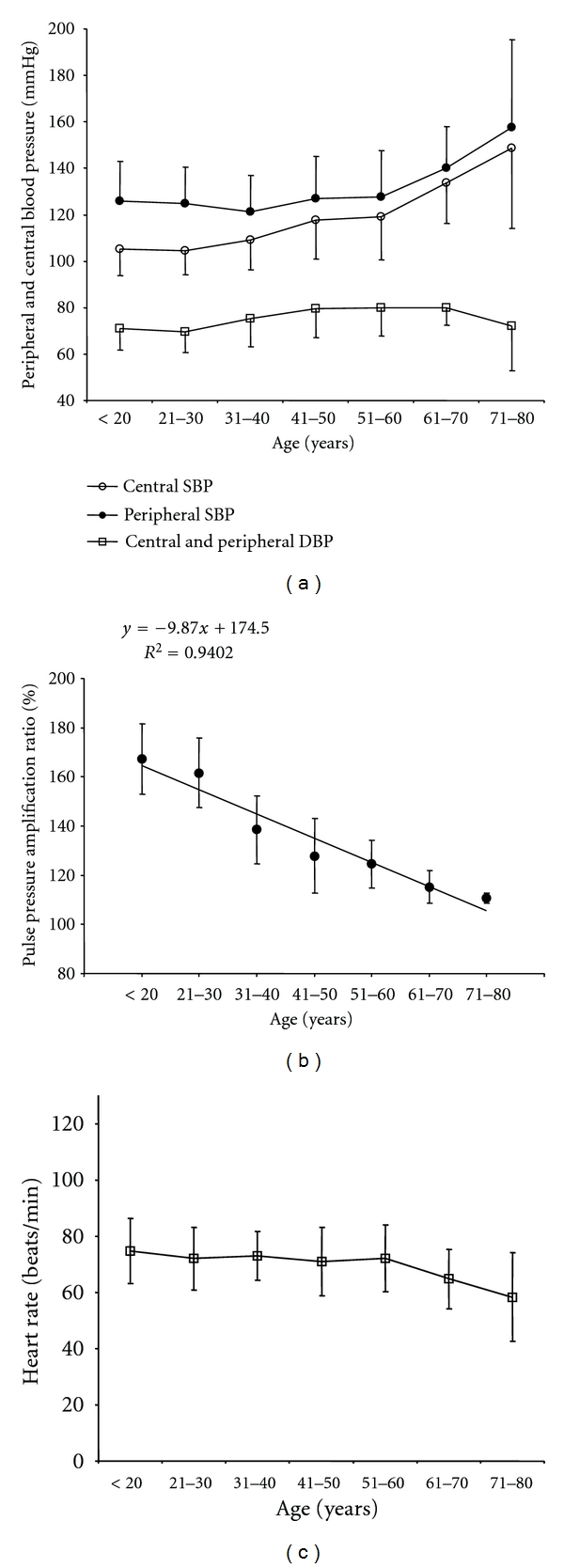
Peripheral (radial) and central (aortic) systolic and diastolic blood pressure (a), pulse pressure amplification ratio (b), and heart rate (c) age- (decade-) related profiles.

As can be seen in Figure 2, central and peripheral systolic blood pressure increased with age, with the steeper increase beyond fifty years. In addition, note the reduction in the centre-periphery systolic pressure amplification with age, reaching stable values from the sixth decade (Figure 2). On the other hand, diastolic pressure showed lesser changes with age, but it definitely decreased beyond sixty years. Heart rate showed a tendency to decrease with age, with major changes over fifty years (Figure 2).

**Figure 3:**
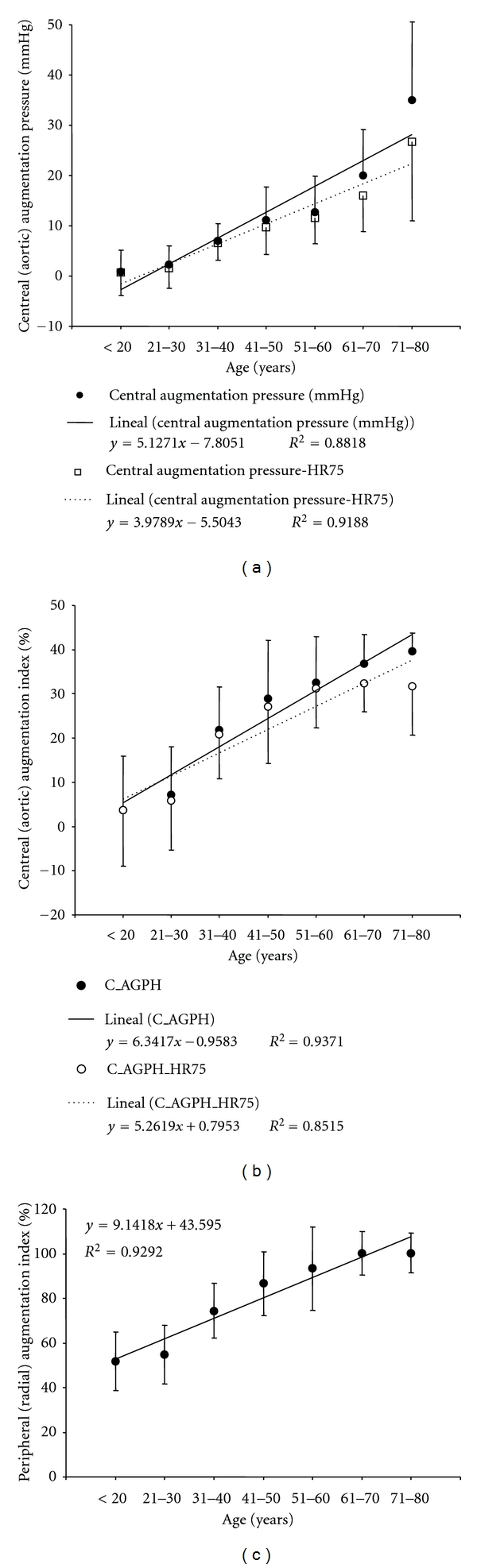
Peripheral (a) and central (b and c) wave reflections indexes age- (decade-) related profiles.

Central augmentation pressure and index levels were higher in older subjects. However, the increase differed, depending on the ages considered. For instance, the major changes in augmentation index were observed between the third and fourth decades (Figure 3). A similar behavior was observed when the peripheral (radial) augmentation index was analyzed (Figure 3).

### 3.2. Common (CCA), Internal (ICA), and External (ECA) Carotid Arteries Plaque Prevalence: CCA Intima-Media Thickness (CIMT) and Diameters

**Figure 4:**
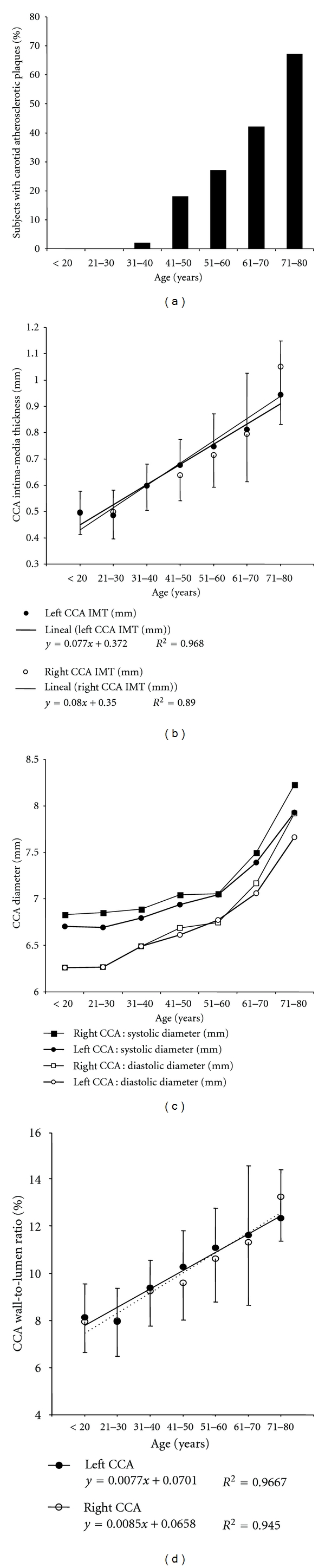
Carotid atherosclerotic plaque prevalence (a) and common carotid artery (CCA) intima-media thickness (b), diameters (c), and wall-to-lumen ratio (d) age- (decade-) related profiles.

Atherosclerotic plaques prevalence increased with age (Figure 4). About this, it is noteworthy that while 2% of subjects between 31–40 years showed plaques, their prevalence in subjects between the 7th and 8th decade was 67% (Figure 4). Starting in the 4th decade, the relationship decade of life—percentage of subjects with carotid plaques—showed a linear relationship: Plaque prevalence (%) = 15.3 ∗ (decade) – 14.6; *R* ^2^ = 0.97.

Figure 4, shows the structural changes in CCA with aging. Note that in both, left and right carotids, the diameter and wall thickness (CIMT) increased with age. However, the thickness changes were larger than those in diameter, resulting in an increase in the wall-to-lumen ratio with age (Figure 4).

### 3.3. CCA Stiffness (Percentual Pulsatility, Compliance, Distensibility, and Stiffness Index)

**Figure 5:**
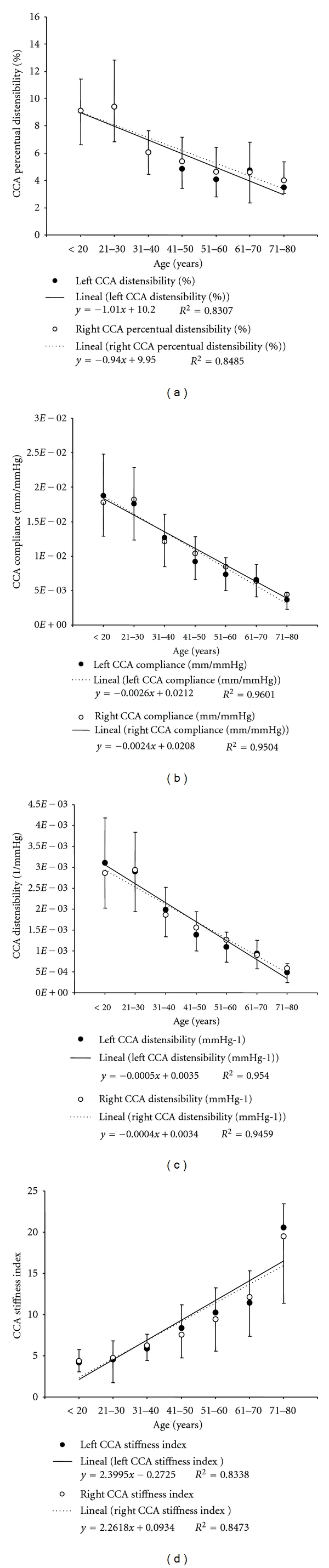
Common carotid artery (CCA) local stiffness parameters. CCA percentual or fractional pulsatility (a), compliance (b), distensibility (c), and Beta or stiffness index (d) age- (decade-) related profiles.

Both left and right CCAs showed a steady increase in stiffness with aging that was evidenced by an increase in the stiffness index and a reduction in compliance, distensibility, and percentual pulsatility (Figure 5).

### 3.4. Aortic Stiffness (Carotid-Femoral Pulse Wave Velocity)

**Figure 6:**
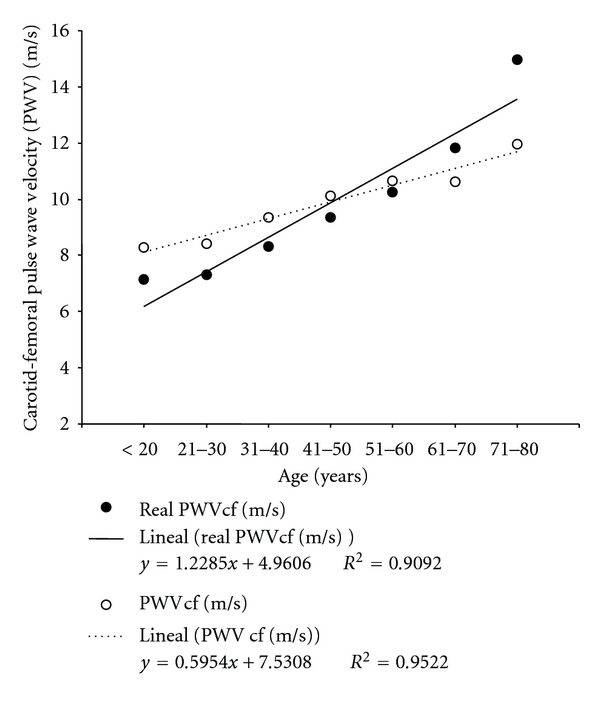
Measured and “real” carotid-femoral pulse wave velocity (PWVcf) age- (decade-) related profiles.

As can be seen in Figure 6, the studied population showed the expected increase in the aortic regional stiffness (PWV increase) with age.

## 4. Discussion

Despite atherosclerosis is not a normal aging change, but a disease condition quite different from true vascular aging, when talking about vascular aging in humans, the analysis is frequently referred to atherosclerosis and its prevalence with age [14, 15]. Furthermore, the study of humans aging has had difficulties in separating aging process from concomitant disease and/or in defining normality and abnormality in the aging process. About this, aging associates structural and functional changes evidenced in variations in vascular parameters that are modified during atherosclerosis and have been proposed as early markers of disease. That stated above could contribute to explain the controversial and/or limited data about age-related cardiovascular changes in different populations. The lack of adequate tools to differentiate the expected (normal) vascular changes due to aging and those related with vascular disease is not a minor issue. About this, it should be kept in mind that for an individual an early diagnosis of vascular disease would be as important as the diagnosis of healthy vascular aging. *In this context, this work has the strength of being the first that uses in Latin America an integrative approach, applied to characterize in a Uruguayan population, the vascular structural and functional changes expected with aging. In turn, the obtained data would be used to define vascular changes not explained by the aging process (abnormal or disease-related vascular changes).*

Several studies have explored the relationship between cardiovascular disease and CIMT in different ethnic populations. The studies' results support the use of CIMT measurement as a surrogate marker of vascular disease, at the time they establish that changes in CIMT could not be considered a disease synonymous [10, 14, 15]. In addition, the studies' results agree in that CIMT increases with advancing age. However, it is noteworthy that among the populations in which CIMT was studied, there were differences in both, the CIMT normal levels and its changes in association with different factors [7, 14, 15]. This issue was recently assessed in the Cardiovascular Risk Factor Multiple Evaluation in Latin America study (CARMELA) [7], which results underlined the differences in age effects across cities.

In this work, and in line with the described changes in CIMT associated with aging, we found an age-related steady increase in the CIMT, in both left and right CCA, with a higher CIMT in the left CCA beyond 40s, in agreement with other authors' findings (Figure 4) [16, 17]. As a whole, our population showed an average CIMT increase of 0.008 mm/year beyond the third decade of life. Then, analyzing our data taking into account those of the CARMELA study, it could be said that the CIMT in the studied Uruguayan population shows similar but not equal levels and age-related changes when compared to other Latin American populations [7]. This is also true when our results are analyzed comparing them with those obtained in other populations. About this, the age-related changes in CIMT found in our population showed a behavior located in the middle between the group in which the variation is large (>0.01 mm/year), consisting of USA [18, 19], central Europe [20–22] and Nordic countries [23], and that with minor age effects, which comprises Spain [24], France [23, 25], and Japan [26, 27]. Taking into account that discussed above, and as was previously suggested, since CIMT levels and changes would differ among regions and ethnic groups, the CIMT distribution in a particular population must be defined prior to its use in cardiovascular diagnosis and/or risk stratification [7]. As will be discussed, the necessity of a population-based determination of normal/reference values is also applicable for other vascular parameters, if an adequate vascular evaluation is to be done.

The age-related vascular structural changes found in our population also included an increase in both systolic and diastolic vascular diameters (Figure 4). It should be noted that although we observed a steady diameter dilatation with age, it was steeper beyond the sixth decade. Changes in central arteries diameter with aging have been previously described and associated with changes in the media layer of the arterial wall (media disorganization and elastic fibers degeneration) [14, 15, 28]. However, the major increase in wall thickness (CIMT) compared with that of the diameter resulted in an age-related increase in the wall to lumen ratio (Figure 4). Analyzing the meaning of the CCA “structural remodeling” found in our population, in the context of the hemodynamic (i.e., pressure changes) variations it showed in association with aging, was beyond this work aims and should be assessed in future works. Anyway, it should be mentioned that we found a reduction in the CCA circumferential stress with age (data not published).

Blood pressure changes with age have been evaluated in several studies and it has been described that systolic blood pressure increases with advancing age [14, 15]. Anyway, age-related pressure variations would not be uniform and would show differences depending on the population characteristics [14, 15]. On the other hand, little changes have been described in diastolic pressure at least until age 60, beyond which a defined reduction in diastolic pressure has been described [14, 15, 29]. Our population showed the expected age-related changes in systolic and diastolic pressure, for both central (aortic) and peripheral (radial) pressure levels (Figure 2). Furthermore, it is noteworthy that the systolic (central and peripheral) pressure variations showed a steeper increase beyond the sixth decade. In addition, beyond that age, the centre-periphery systolic amplification remained constant, which is in agreement with previous works [14, 15, 30].

Pressure changes have been associated with changes in vascular stiffness. About this, manifestations of arterial stiffness include its effects on the arterial pressure levels and wave [31]. In addition, it should be noted that vascular (mainly at the aortic and central arteries levels) stiffening has been described as a true aging change; a fact not accepted by some authors and that would vary depending on the subjects characteristics [14, 15]. In this context, we found an increase in waves' reflections (evidenced by central and peripheral wave reflection parameters) with age (Figure 3) and that both the CCA and the aorta progressively stiffen with aging, as was evidenced by the CCA distensibility and compliance reduction and by the increase in the CCA stiffness index (Figure 5) and PWV (Figure 6). These findings agree with published data [14, 15, 32, 33].

In agreement with our findings, recently Boutouyrie and Vermeersch [3] performed an important study in which PWV recordings from 16.867 subjects from 13 different centers across eight European countries were collected. Among the included subjects, those (*n* = 11.092) free from overt cardiovascular disease, nondiabetic, and untreated by either antihypertensive or lipid-lowering drugs constituted the reference value population. Subjects were categorized by age decade and further subdivided according to BP categories. From the authors' data, regression lines for mean “real” PWV, considering the subjects blood pressure levels were: PWV (m/s) = 0.97 ∗ decade + 4.26 (optimal pressure), PWV (m/s) = 1.20 ∗ decade + 4.02 (normal pressure), PWV (m/s) =1.18 ∗ decade + 4.46 (high normal pressure), and PWV (m/s) = 1.37 ∗ decade + 5.79 (grade 1 HTA). In our population of nonhypertensive patients, the relationship was PWV (m/s) = 1.22 ∗ decade + 4.96 (Figure 6). Then, our population showed a rate of PWV increase (0.12 m/s per year) that would be comparable to that found in by Boutouyrie and Vermeersch for nonhypertensive subjects. On the other hand, it is to note that in average, the subjects included in our study showed higher PWV levels than those included in Boutouyrie and Vermeersch work [3]. The meaning of the differences in PWV, and the mechanisms that could explain them should be analyzed in future works. Anyway, those differences, add support to that mentioned above related with the importance of obtaining population-based normal/reference values to perform a correct interpretation of a subject vascular evaluation.

Finally, it would be worth mentioning that we found an aging increase in the prevalence of atherosclerotic plaques (Figure 4). Such age-related increase in the number of atheroma plaques is in agreement with the described augmented prevalence of atherosclerosis with age [14, 15]. In particular, it is noteworthy that for both, males and females, Joakimsen et al. [34] described and increase in the number of plaques diagnosed from the fourth until the ninth decade. Furthermore, in agreement with our findings, the authors described that the predilection site of atherosclerosis was the bifurcation segment of the carotid artery, for both sexes at any age [34]. If there are age-related differences in the plaques characteristics (biomechanical and/or structural properties) would be an interesting issue to be assessed in future works.

Finally, it is noteworthy that future works are necessary to analyze how cardiovascular risk factors impact on the expected changes associated with aging and to determine normal and reference values for the different vascular parameters. In turn, that should be done taking into account possible gender differences.

## 5. Conclusions

Age-related structural and functional vascular parameters profiles were obtained in the context of the CUiiDARTE Project, using gold standard methodologies and techniques, for a Uruguayan asymptomatic population, and analyzed taking into account available data from other populations.

The work has the strength of being the first in Latin America that applies an integrative approach to characterize age-related structural (i.e., CIMT, Diameter, wall-to-lumen ratio) and functional (i.e., CCA stiffness, aortic stiffness, peripheral, and central pressure waveform indexes) parameters using gold standard techniques.

The obtained data could be used in the vascular diagnosis to define/differentiate normal changes and abnormal or disease-related vascular variations.

## Figures and Tables

**Table 1:** Anthropometric and biochemical measurements of the seven different age groups.

	≤20 years	21–30 years	31–40 years	41–50 years	51–60 years	61–70 years	71–80 years
	MV ± SD	MV ± SD	MV ± SD	MV ± SD	MV ± SD	MV ± SD	MV ± SD
*N*	74	79	43	50	59	50	33
Gender (male, %)	44	49	38	44	51	39	46
Age [years]	19.1 ± 0.2	24.8 ± 1.1	35.0 ± 2.7	45.7 ± 3.4	56.4 ± 4.0	64.3 ± 4.9	74.0 ± 3.3
Body height (cm)	167.1 ± 8.3	166.3 ± 8.2	163.1 ± 6.5	166.4 ± 10.5	159.4 ± 4.3	166.0 ± 15.1	158.0 ± 8.1
Body weight (Kg)	59.4 ± 7.6	59.9 ± 8.4	56.1 ± 6.8	63.3 ± 10.5	56.6 ± 6.5	65.7 ± 10.0	62.0 ± 8.1
Body mass index (Kg/m^2^)	21.3 ± 1.8	21.7 ± 4.1	21.1 ± 2.1	22.8 ± 2.2	22.3 ± 2.4	23.8 ± 0.8	24.8 ± 1.0
Total cholesterol (mg/dL)	184.8 ± 34.1	175.2 ± 24.9	190.2 ± 21.6	184.9 ± 18.3	219.4 ± 8.9	190.5 ± 21.8	183.0 ± 23.1
HDL-cholesterol (mg/dL)	63.5 ± 7.9	64.6 ± 13.8	68.0 ± 27.0	67.5 ± 6.4	73.0 ± 19.5	66.0 ± 10.2	64.0 ± 9.2
LDL-cholesterol (mg/dL)	102.0 ± 21.0	92.0 ± 19.2	111.7 ± 29.6	100.5 ± 6.4	128.7 ± 12.4	92.0 ± 12.2	90.0 ± 13.2
Triglycerides (mg/dL)	96.5 ± 47.8	85.4 ± 27.4	58.3 ± 20.5	57.5 ± 2.1	60.3 ± 20.8	64.0 ± 18.1	80.0 ± 5.1
Total cholesterol/HDL cholesterol	2.9 ± 0.3	2.8 ± 0.6	3.0 ± 1.1	2.7 ± 0.1	3.1 ± 0.9	2.9 ± 0.2	2.8 ± 0.1
Non-HDL cholesterol	121.3 ± 27.5	110.5 ± 22.8	123.7 ± 32.3	112.0 ± 7.1	140.7 ± 16.8	124 ± 17.3	119.3 ± 19.3
Glycaemia (mg/dL)	79.3 ± 2.2	84.1 ± 5.9	69.2 ± 16.5	74.0 ± 19.1	76.0 ± 15.8	72.5 ± 6.4	82.0 ± 12.1
Family history of premature CVD [%]	3	3	4	3	3	5	5

Mean (MV) ± standard deviation (SD). HDL and LDL: high and low density lipoprotein cholesterol, respectively. *N*: number of subjects. CVD: cardiovascular disease.

## References

[1] Naghavi M, Falk E, Hecht HS (2006). From vulnerable plaque to vulnerable patient-part III: executive summary of the screening for heart attack prevention and education (SHAPE) task force report. *American Journal of Cardiology*.

[2] Greenland P, Alpert JS, Beller GA (2010). 2010 ACCF/AHA guideline for assessment of cardiovascular risk in asymptomatic adults: a report of the American college of cardiology foundation/American heart association task force on practice guidelines. *Journal of the American College of Cardiology*.

[3] Boutouyrie P, Vermeersch SJ (2010). Determinants of pulse wave velocity in healthy people and in the presence of cardiovascular risk factors: establishing normal and reference values. *European Heart Journal*.

[4] Escobedo J, Schargrodsky H, Champagne B (2009). Prevalence of the metabolic syndrome in Latin America and its association with sub-clinical carotid atherosclerosis: the CARMELA cross sectional study. *Cardiovascular Diabetology*.

[5] Champagne BM, Sebrié EM, Schargrodsky H, Pramparo P, Boissonnet C, Wilson E (2010). Tobacco smoking in seven Latin American cities: the CARMELA study. *Tobacco Control*.

[6] Vinueza R, Boissonnet CP, Acevedo M (2010). Dyslipidemia in seven Latin American cities: CARMELA study. *Preventive Medicine*.

[7] Touboul PJ, Vicaut E, Labreuche J (2010). Common carotid artery intima-media thickness: the cardiovascular risk factor multiple evaluation in Latin America (CARMELA) study results. *Cerebrovascular Diseases*.

[8] Friedewald WT, Levy RI, Fredrickson DS (1972). Estimation of the concentration of low-density lipoprotein cholesterol in plasma, without use of the preparative ultracentrifuge. *Clinical Chemistry*.

[9] National Cholesterol Education Program (NCEP) Expert Panel on Detection (2002). Third report of the national cholesterol education program (NCEP) expert panel on detection, evaluation, and treatment of high blood cholesterol in adults (adult treatment panel III) final report. *Circulation*.

[10] Stein JH, Korcarz CE, Hurst RT (2008). Use of carotid ultrasound to identify subclinical vascular disease and evaluate cardiovascular disease risk: a consensus statement from the American society of echocardiography carotid intima-media thickness task force endorsed by the society for vascular medicine. *Journal of the American Society of Echocardiography*.

[11] Laurent S, Cockcroft J, Van Bortel L (2006). Expert consensus document on arterial stiffness: methodological issues and clinical applications. *European Heart Journal*.

[12] Bia D, Zócalo Y, Armentano R (2009). Non-invasive biomechanical evaluation of implanted human cryopreserved arterial homografts: comparison with pre-implanted cryografts and arteries from human donors and recipients. *Annals of Biomedical Engineering*.

[13] Pauca AL, O’Rourke MF, Kon ND (2001). Prospective evaluation of a method for estimating ascending aortic pressure from the radial artery pressure waveform. *Hypertension*.

[14] Nichols WW, O’Rourke M, Nichols WW, O’Rourke M (2005). Properties of the arterial wall: theory. *Mc Donald’s Blood Flow in Arteries: Theoretical, Experimental and Clinical Principles*.

[15] Nichols WW, O’Rourke M, Nichols WW, O’Rourke M (2005). Properties of the arterial wall: practice. *Mc Donald’s Blood Flow in Arteries: Theoretical, Experimental and Clinical Principles*.

[16] Touboul PJ, Labreuche J, Vicaut E (2009). Country-based reference values and impact of cardiovascular risk factors on carotid intima-media thickness in a french population: the “Paroi Artérielle et risque cardio-vasculaire” (PARC) study. *Cerebrovascular Diseases*.

[17] Luo X, Yang Y, Cao T, Li Z (2011). Differences in left and right carotid intima-media thickness and the associated risk factors. *Clinical Radiology*.

[18] O’Leary DH, Polak JF, Kronmal RA, Manolio TA, Burke GL, Wolfson SK (1999). Carotid-artery intima and media thickness as a risk factor for myocardial infarction and stroke in older adults: cardiovascular health study. *The New England Journal of Medicine*.

[19] Chambless LE, Heiss G, Folsom AR (1997). Association of coronary heart disease incidence with carotid arterial wall thickness and major risk factors: the atherosclerosis risk in communities (ARIC) study, 1987–1993. *American Journal of Epidemiology*.

[20] Allan P, Mowbray P, Lee A, Fowkes F (1997). Relationship between carotid intima-media thickness and symptomatic and asymptomatic peripheral arterial disease: the edinburgh artery study. *Stroke*.

[21] Ebrahim S, Papacosta O, Whincup P (1999). Carotid plaque, intima media thickness, cardiovascular risk factors, and prevalent cardiovascular disease in men and women: the British regional heart study. *Stroke*.

[22] Van der Meer IM, Bots M, Hofman A, del Sol AI, Van der Kuip D, Witteman J (2004). Predictive value of noninvasive measures of atherosclerosis for incident myocardial infarction: the rotterdam study. *Circulation*.

[23] Zureik M, Touboul PJ, Bonithon-Kopp C (1999). Cross-sectional and 4-year longitudinal associations between brachial pulse pressure and common carotid intima-media thickness in a general population: the EVA study. *Stroke*.

[24] Junyent M, Gilabert R, Núñez I (2005). Carotid ultrasound in the assessment of preclinical atherosclerosis. Distribution of intima-media thickness values and plaque frequency in a Spanish community cohort. *Medicina Clinica*.

[25] Ferrieres J, Elias A, Ruidavets JB (1999). Carotid intima-media thickness and coronary heart disease risk factors in a low-risk population. *Journal of Hypertension*.

[26] Ando F, Takekuma K, Niino N, Shimokata H (2000). Ultrasonic evaluation of common carotid intima-media thickness (IMT). Influence of local plaque on the relationship between IMT and age. *Journal of Epidemiology*.

[27] Homma S, Hirose N, Ishida H, Ishii T, Araki G (2001). Carotid plaque and intima-media thickness assessed by B-mode ultrasonography in subjects ranging from young adults to centenarians. *Stroke*.

[28] Virmani R, Avolio AP, Mergner WJ (1991). Effect of aging on aortic morphology in populations with high and low prevalence of hypertension and atherosclerosis: comparison between occidental and Chinese communities. *American Journal of Pathology*.

[29] Franklin SS, Gustin W, Wong ND (1997). Hemodynamic patterns of age-related changes in blood pressure: the Framingham heart study. *Circulation*.

[30] Avolio A (2008). Central aortic blood pressure and cardiovascular risk: a paradigm shift?. *Hypertension*.

[31] O’Rourke MF, Staessen JA, Vlachopoulos C, Duprez D, Plante GE (2002). Clinical applications of arterial stiffness; definitions and reference values. *American Journal of Hypertension*.

[32] Segers P, Mahieu D, Kips J (2009). Amplification of the pressure pulse in the upper limb in healthy, middle-aged men and women. *Hypertension*.

[33] Chung JW, Lee YS, Kim JH (2010). Reference values for the augmentation index and pulse pressure in apparently healthy Korean subjects. *Korean Circulation Journal*.

[34] Joakimsen O, Bønaa KH, Stensland-Bugge E, Jacobsen BK (1999). Age and sex differences in the distribution and ultrasound morphology of carotid atherosclerosis: the Tromso study. *Arteriosclerosis, Thrombosis, and Vascular Biology*.

